# Auraptene in the Peels of *Citrus kawachiensis* (Kawachi Bankan) Ameliorates Lipopolysaccharide-Induced Inflammation in the Mouse Brain

**DOI:** 10.1155/2014/408503

**Published:** 2014-05-15

**Authors:** Satoshi Okuyama, Kana Yamamoto, Hirotomo Mori, Nobuki Toyoda, Morio Yoshimura, Yoshiaki Amakura, Takashi Yoshida, Kuniaki Sugawara, Masahiko Sudo, Mitsunari Nakajima, Yoshiko Furukawa

**Affiliations:** ^1^Department of Pharmaceutical Pharmacology, College of Pharmaceutical Sciences, Matsuyama University, 4-2 Bunkyo-cho, Matsuyama, Ehime 790-8578, Japan; ^2^Department of Pharmacognosy, College of Pharmaceutical Sciences, Matsuyama University, 4-2 Bunkyo-cho, Matsuyama, Ehime 790-8578, Japan; ^3^Department of Planning and Development, Ehime Beverage Inc., 478 Anjyoji, Matsuyama, Ehime 791-8603, Japan

## Abstract

Examination of the dried peel powder of *Citrus kawachiensis*, one of the citrus products of Ehime, Japan, showed that it contained naringin (NGIN; 44.02 ± 0.491 mg/g), narirutin (NRTN; 4.46 ± 0.0563 mg/g), auraptene (AUR; 4.07 ± 0.033 mg/g), and 3,5,6,7,8,3′,4′-heptamethoxyflavone (HMF; 0.27 ± 0.0039 mg/g). When this dried peel powder was orally preadministered at the dose of 1.2 or 2.4 g/kg/day for 7 days into lipopolysaccharide- (LPS-) injected mice, an animal model of systemic inflammation, it suppressed (1) LPS-induced loss of body weight and abnormal behavior in the open field, (2) LPS-induced activation of microglia and astrocytes in the hippocampus, and (3) LPS-induced expression of cyclooxygenase (COX)-2, which were coexpressed in astrocytes of these mice. When NGIN or AUR was preadministered to LPS-injected mice at an amount similar to that in the peel powder, AUR, but not NGIN, had the ability to suppress the LPS-induced inflammation in the brain of these model mice. The dried powder of flavedo tissue (the outer colored layer of the mesocarp of a citrus fruit) and juice, which contained sufficient amounts of AUR, also had anti-inflammatory effect. These results suggest that AUR was the main ingredient responsible for the anti-inflammatory property of the dried peels of *C. kawachiensis*.

## 1. Introduction

**Figure 1:**
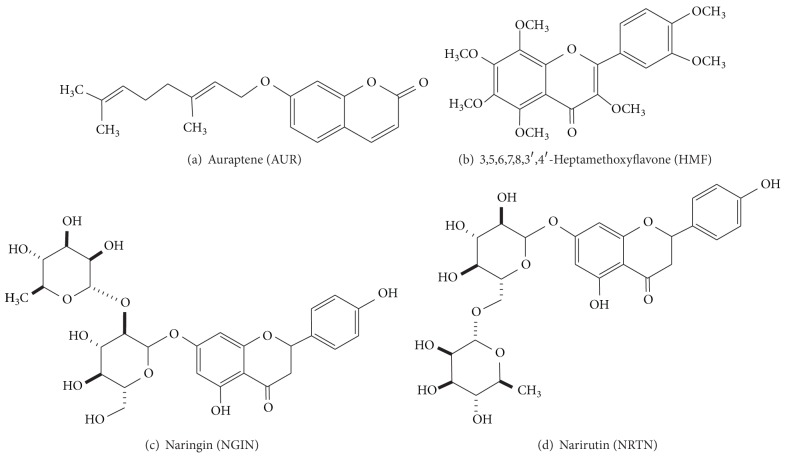
Structures of compounds detected in peel powder of* C. kawachiensis* (kawachi bankan).

Inflammation is a pathophysiological phenomenon that is involved in numerous diseases of not only peripheral tissues but also the central nervous system (CNS). For the example, one of the representative consequences of ischemia is inflammation in the brain [1]. After ischemic onset, inflammatory cells such as blood-derived leukocytes and microglia are activated and accumulate within the brain tissue, subsequently leading to inflammatory injury. In the processes of ischemia/stroke, the molecular cues generated by cerebral ischemia activate components of the innate immunity system, promote inflammatory signaling, and contribute to tissue damage [2]. We recently reported, by using an ischemic mouse model, that subcutaneously (*s.c.*) administered auraptene (AUR; Figure 1(a)), a* Citrus* coumarin, effectively inhibits microglia activation, cyclooxygenase-2 (COX-2) expression by astrocytes, and neuronal cell death in the hippocampus [3]. We also observed that the peels of* Citrus kawachiensis* (kawachi bankan), which is one of the citrus products of Ehime, Japan, contain AUR [4] in higher content than found in any other* Citrus* peels [5]. These results suggested that the peels of* C. kawachiensis* might be a good source of AUR for suppressing inflammation-induced damage in the CNS.

Therefore, the objective of this study was to ascertain the effectiveness of orally administered peel powder of* C. kawachiensis* against inflammation in the brain. As a sample, the peel powder of* C. kawachiensis* was prepared by adopting the processes of drying and sieving. As a model animal of systemic inflammation, we used mice that had been intraperitoneally (*i.p.*) administered lipopolysaccharide (LPS), as peripheral injected LPS has the ability to induce immune responses in the brain [6].

## 2. Materials and Methods

### 2.1. Preparation of Citrus Materials and Chemicals

Fruits of* C. kawachiensis* were harvested in Yawatahama, Ehime, Japan. For the preparation of dried peels, the peels of the fruits (1555 g) after having been squeezed to obtain the juice were chopped into small pieces and dried* in vacuo* at 60°C for 1 day. The dried peels (*ca*. 300 g) were then milled and ground to a fine powder with a mill mixer (Iwatani IFM-660DG, Tokyo, Japan). The powder, after having been passed through a 150-mesh sieve, was used as the test sample. For the preparation of the dried flavedo and the dried albedo, flavedo tissue (exterior yellow peel) and albedo tissue (interior white peel) were manually separated from fresh fruit with a small knife and dried* in vacuo* at 60°C for 1 day. For the preparation of the dried juice, the juice was freeze-dried. These materials (dried flavedo, dried albedo, and dried juice) were then mechanically ground to a fine powder with the mill mixer and filtered through a 150-mesh sieve as in the case of the dried peels. Naringin (NGIN; Figure 1(c)) and AUR were obtained from LKT Laboratories (St. Paul, MN, USA).

### 2.2. Determination of Flavonoid Contents in Samples

Dried* Citrus* samples (1 g) were extracted by sonication for 20 min in ethanol (30 mL) at room temperature. The mixture was then centrifuged at 10,000 ×g for 5 min, and the resulting supernatant layer was analyzed by reversed-phase HPLC. Standard solutions of NGIN, narirutin (NRTN; Figure 1(d)), heptamethoxyflavone (HMF; Figure 1(b), and AUR were prepared in methanol at a concentration of 1.0 mg/mL. Stock solutions were diluted to prepare a calibration curve with linearity in the range of 0.01~1.0 mg/mL. Each value was reported as the mean of 3 analyses. HPLC analysis was carried out by using a Shimadzu Prominence system (Shimadzu, Kyoto, Japan). HPLC conditions were as follows: column, L-column ODS (5 *μ*m, 150 × 2.1 mm i.d.; Chemicals Evaluation and Research Institute, Tokyo, Japan); mobile phase, solvent A (5% acetic acid) and solvent B (acetonitrile): 0~30 min, 0~50% B in A; 30~35 min, 50~85% B in A; 35~40 min, 85~85% B in A; injection volume, 2 *μ*L; column temperature, 40°C; flow rate, 0.3 mL/min; detection, flavonoids at 280 nm and AUR at 320 nm. NRTN was obtained from ChromaDex (Irvine, CA, USA). HMF was prepared from commercial orange oil (Wako, Osaka, Japan) as described previously [4].

### 2.3. Animals

Nine-week-old male C57BL/6 strain mice were purchased from Japan SLC (Hamamatsu, Japan). Mice in all groups were kept at 23 ± 1°C under a 12 h light/dark cycle (light on 8:00~20:00). During the experimental period, the mice were given free access to tap water and food until 08:30 and then deprived of food until the time of administration of samples or vehicle (16:00). All animal experiments were carried out in accordance with the guidelines for animal experimentation specified by the Animal Care and Use Committee of Matsuyama University.

### 2.4. Administration of Citrus Samples

For experiments, mice weighing about 25 g were randomized into 4 groups. The dried test samples were dissolved in distilled water. In the 2 test-sample groups, mice were administered sample solution (0.75 mL) of 2 different doses* per os* (*p.o*.) once a day for 7 days. Mice in the other 2 groups (nontreated group: *n* = 6 and LPS-treated group: *n* = 9) were administered vehicle (distilled water) in the same way. The dose for each test sample-administered group was as follows: 1.2 g of dried peels/kg/day (*n* = 9); 2.4 g of dried peels/kg/day (*n* = 9); 1.2 g of dried flavedo/kg/day (*n* = 8); 2.4 g of dried flavedo/kg/day (*n* = 8); 100 g of dried juice/kg/day (*n* = 6); 200 g of dried juice/kg/day (*n* = 6).

### 2.5. Administration of NGIN or AUR

Mice, weighing about 25 g, were randomized into 6 groups. NGIN and AUR were separately dissolved in DMSO/polyethylene glycol (PEG) 300 (1 : 1) solutions as previously mentioned [3]. The animals in 4 groups were subcutaneously administered these solutions (125 *μ*L) to achieve 50 mg of NGIN/kg/day (*n* = 5), 100 mg of NGIN/kg/day (*n* = 5), 10 mg of AUR/kg/day (*n* = 5), or 25 mg of AUR/kg/day (*n* = 5), once a day for 7 days. Mice of the other 2 groups (nontreated group: *n* = 5 and LPS-treated group: *n* = 5) were administered vehicle (DMSO/PEG) in the same way.

### 2.6. LPS Treatment

LPS (from* Salmonella enterica *serotype typhimurium) was purchased from Sigma-Aldrich (St. Louis, MO, USA) and dissolved with saline. On the seventh day after the administration of* Citrus* samples, 25 *μ*g of LPS in 0.125 mL of saline was immediately intraperitoneally (*i.p.*) administered (1 mg/kg of mouse) to the LPS-treated group and test-sample groups. For the nontreated group, vehicle (0.125 mL of saline) was* i.p.* administered.

### 2.7. Open-Field Test

One day after the LPS injection, mice were weighed and submitted to a test of abnormal behavior. Abnormal activity was evaluated by using the open-field test. Each mouse was placed in the center of an open-field apparatus (W70 × D70 × H50 cm; center 35 × 35 cm), and its behavior was analyzed for 10 min with the ANY-maze Video Tracking System (Stoelting, Wood Dale, IL, USA), which was connected to a USB digital camera.

### 2.8. Immunohistochemistry

After the tests of abnormal behavior, mice were anesthetized and transcardially perfused with ice-cold PBS. Their brains were then removed and processed for optical microscopy or confocal fluorescence microscopy as previously reported [6]. For optical microscopy, a rabbit polyclonal antibody against ionized calcium-binding adaptor molecule 1 (IBA1; Wako, Osaka, Japan) was used as the primary antibody. The secondary antibody was EnVision-plus system HRP-labeled polymer (anti-rabbit; Dako, Glostrup, Denmark). Immunoreactivity was developed and visualized by use of DAB substrate (SK-4100; Vector Laboratories, Burlingame, CA, USA) and quantified by using ImageJ software (NIH, Bethesda, MD, USA). For confocal fluorescence microscopy, the primary antibodies used were goat anti-COX-2 (Santa Cruz Biotechnology, Santa Cruz, CA, USA) and mouse anti-glial fibrillary acidic protein (GFAP; Sigma-Aldrich, St. Louis, MO, USA); and the secondary antibodies were Alexa Fluor 488-labeled donkey anti-goat IgG (H + L) (Invitrogen, Carlsbad, CA, USA) and Alexa Fluor 568-labeled goat anti-mouse IgG (H + L), respectively. The mounting medium used was VECTASHIELD (Vector Laboratories, Burlingame, CA, USA). Images of the hippocampus were captured with a confocal fluorescence microscopy system (LSM510; Zeiss, Oberkochen, Germany).

### 2.9. Statistical Analysis

Data for the individual groups were expressed as means ± SEM. Data were analyzed by one-factor ANOVA followed by Bonferroni's multiple comparison test (Prism 5; GraphPad Software, La Jolla, CA, USA). Significance was defined as *P* < 0.05.

## 3. Results

### 3.1. Contents of Main Compounds in the Dried Peels of* C. kawachiensis*

**Figure 2:**
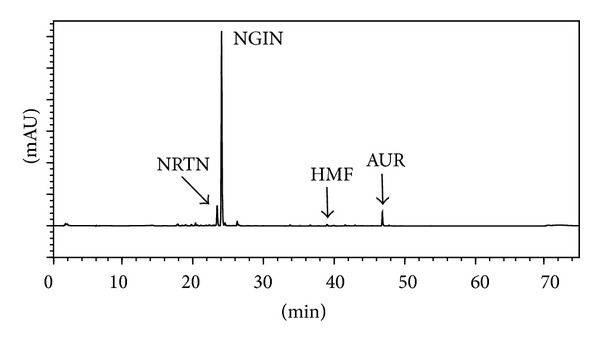
HPLC profile (280 nm) of peel powder of* C. kawachiensis*.

The contents of 4 selected compounds (AUR, HMF, NRTN, and NGIN) in the dried peels were quantified by an absolute calibration method with reference to the peak areas on the HPLC chromatogram. The chromatogram of peel powder of* C. kawachiensis* is depicted in Figure 2. As shown in Table 1, the primary compound was NGIN, at 44.02 ± 0.491 mg/g of the sample. NRTN, AUR, and HMF contents were much lower, being 4.46 ± 0.0563 mg/g, 4.07 ± 0.033 mg/g, and 0.27 ± 0.0039 mg/g, respectively.

### 3.2. Effect of the Dried Peels of* C. kawachiensis* on LPS-Induced Sickness Response

Mice were pretreated with sample (dried peels) or vehicle (distilled water) once daily for 7 days followed by a single* i.p.* injection of LPS or vehicle (saline). In the present study, the dose of the dried peel powder was designed to be 1.2 g/kg/day or 2.4 g/kg/day. The amounts of AUR, HMF, NRIN, and NRTN contained in these sample solutions are shown in Table 2.

**Figure 3:**
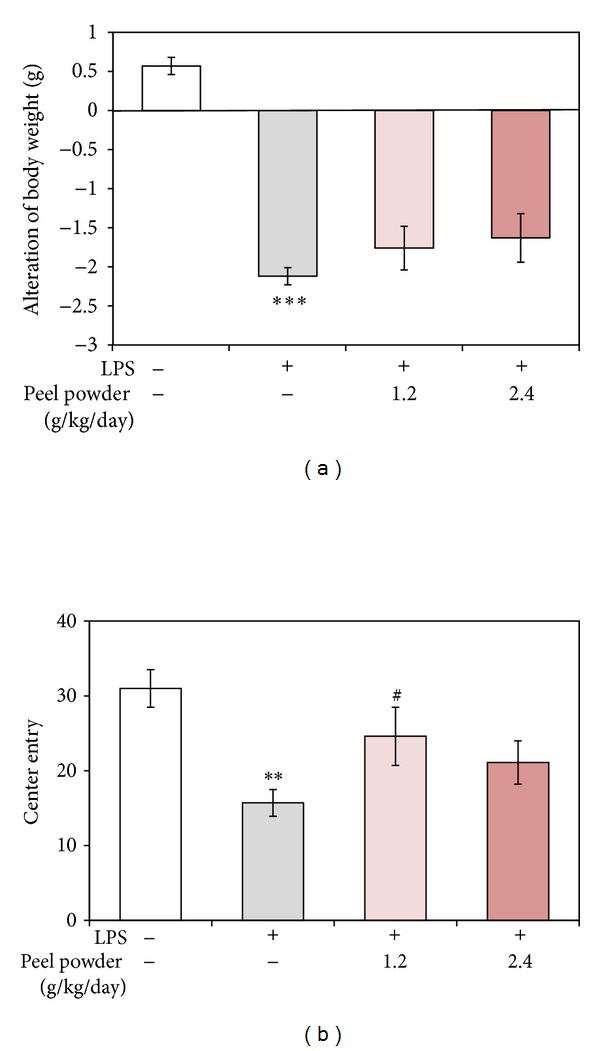
Effect of* p.o.* administration of the dried peels of* C. kawachiensis* on LPS-induced loss of body weight (a) and LPS-induced abnormal behavior (b). Symbols indicate significant differences versus nontreated group (**P < 0.01; ***P < 0.001) and versus LPS-treated group (^#^ *P* < 0.05).

We assessed the changes in body weight loss at 20 h after injection of LPS, as one index to estimate the degree of the LPS-induced sickness response. Previous study showed that* i.p.* administration of LPS (0.1 mg/kg) significantly reduces food intake and body weight at 8 h and 1 day after injection but not at 2~4 days [7].  Figure 3(a) shows that* i.p.* administration of LPS caused a significant reduction in body weight (***P < 0.001). The pretreatment of the animals with the dried peel powder attenuated dose-dependently this LPS-induced reduction in body weight, though this effect was not significant for either 1.2 g/kg/day-treated group (*P* = 0.267) or 2.4 g/kg/day-treated group (*P* = 0.142).

As another index of the LPS-induced sickness response, we examined the LPS-induced abnormal behavior observed in an open-field setting. We measured the frequency of center entry in the open-field test, which is known to be an indicator of behavioral activity [8].  Figure 3(b) shows that the frequency of center entry of the LPS-treated mice (15.7 ± 1.8) was significantly (**P < 0.01) decreased compared with that of nontreated group (31 ± 2.5). This value for 1.2 g/kg/day-treated group (24.6 ± 3.9) was significantly (^#^ *P* < 0.05) greater than that for the LPS-treated group. The value for the higher-dose sample (2.4 g/kg/day-treated group) was also greater (21.1 ± 2.9) than that for the LPS-treated group but not significantly so (*P* = 0.184).

These data indicate that acute activation of the peripheral innate immune system with LPS induced the sickness response in mice and that the peels of* C. kawachiensis* had the ability to suppress the symptoms of it.

### 3.3. Effect of the Dried Peels of* C. kawachiensis* on LPS-Induced Microglial Activation

It is well known that microglia are distributed throughout the brain as a network of immunocompetent cells and that the cells become rapidly activated in response to injury or in the presence of pathogens [9]. Previous studies indicated that peripheral injected LPS induced microglial activation in the brain [6, 10]. So we used antibody against IBA1, a microglial marker, to stain microglia in the hippocampal region of the various groups of mice.

**Figure 4:**
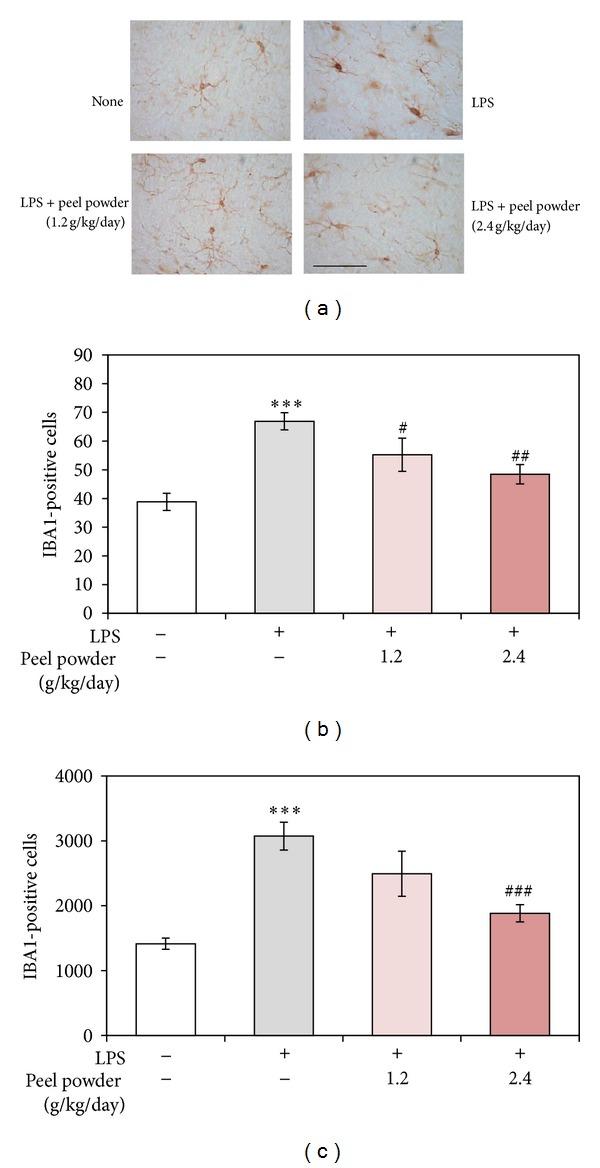
Effect of* p.o.* administration of the dried peels of* C. kawachiensis* on LPS-induced microglial activation. (a) Sagittal sections of the hippocampus were stained with anti-IBA1 antibody. Scale bar shows 50 *μ*m. Results of quantitative analysis of number (b) and the area (c) of IBA1-positive cells by use of ImageJ software are shown. Values are means ± SEM (*n* = 8~10 for each group). Symbols indicate significant differences versus nontreated group (***P < 0.001) and versus LPS-treated group (^#^ *P* < 0.05, ^##^ *P* < 0.01, and ^###^ *P* < 0.001).

In the nontreated group, only a few IBA1-positive cells were observed as being in the ramified form (an inactivated form) in the hippocampus (Figure 4(a)-none). In the LPS-treated group, the number of IBA1-positive cells had increased; and their shape had changed to that of “ameboid microglia” (an activated form; Figure 4(a)-LPS) as previously reported [6]. Quantitative analysis of the number of these cells (Figure 4(b); ***P < 0.001) and the areas of these cells (Figure 4(c); ***P < 0.001) revealed that both had significantly increased compared with those for the nontreated group. In the 1.2 g/kg/day-treated group and 2.4 g/kg/day-treated group, the number of IBA1-positive cells was significantly lower than that in the LPS-treated group (Figures 4(a) and 4(b); ^#^ *P* < 0.05 for 1.2 g/kg/day-treated group and ^##^ *P* < 0.01 for 2.4 g/kg/day-treated group). In the 1.2 g/kg/day-treated group and 2.4 g/kg/day-treated group, the shape of the IBA1-positive cells indicated the inactive ramified form (Figure 4(a)); and the area occupied by these cells was lower than that for the LPS-treated group (Figure 4(c); *P* = 0.076 for the 1.2 g/kg/day-treated group and ^###^ *P* < 0.001 for the 2.4 g/kg/day-treated group). These results indicate that the peels of* C. kawachiensis* had the ability to suppress LPS-induced microglial activation in the brain of the treated mice.

### 3.4. Effect of the Dried Peels of* C. kawachiensis* on LPS-Induced Astroglial Activation and COX-2 Expression

**Figure 5:**
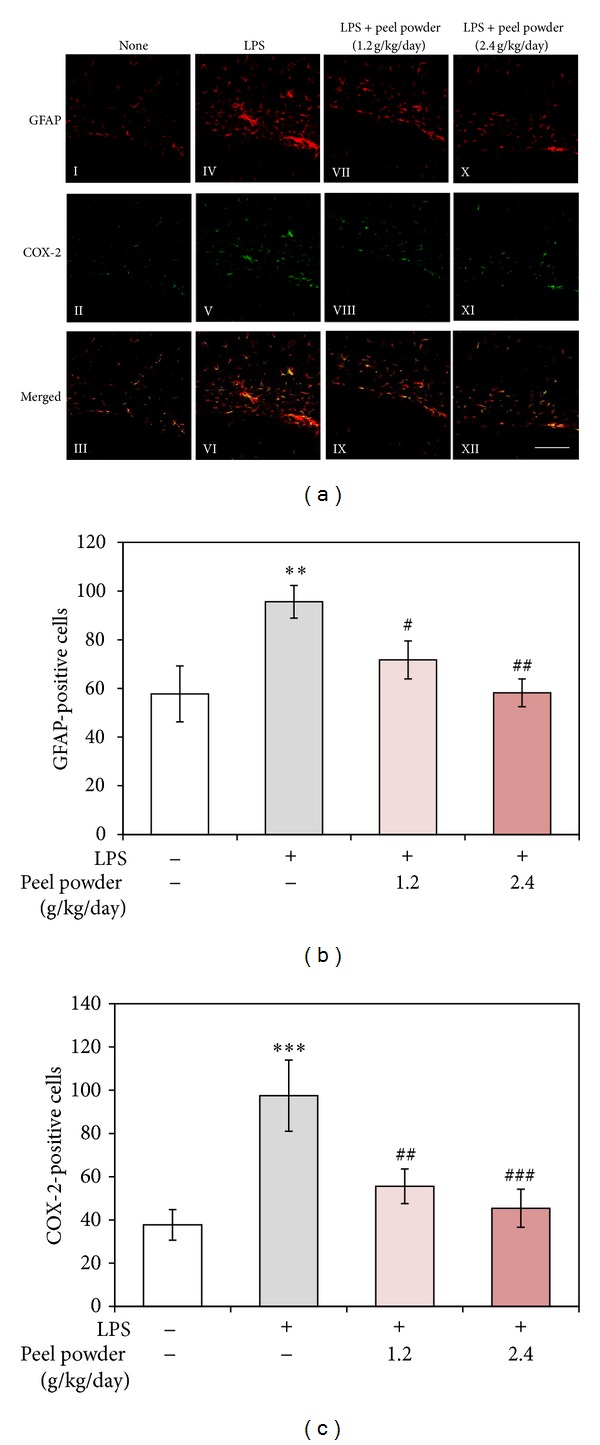
Effect of* p.o.* administration of the dried peels of* C. kawachiensis* on LPS-induced expression of GFAP and COX-2 in the mouse hippocampus. (a) Sagittal sections were stained with antibodies specific for GFAP (red: I, IV, VII, and X) or COX-2 (green: II, V, VIII, and XI). Merged pictures (III, VI, IX, XII) show cells that co-expressed GFAP and COX-2 (yellow cells). Scale bar indicates 100 *μ*m. (b) GFAP-positive cells and (c) COX-2-positive cells were counted by use of ImageJ software. Values are means ± SEM (*n* = 8~10 for each group). Symbols indicate significant differences versus nontreated group (**P < 0.01 and ***P < 0.001) and versus LPS-treated group (^#^ *P* < 0.05, ^##^ *P* < 0.01, and ^###^ *P* < 0.001).

Recent studies have suggested the crucial importance of astrocytes as well as microglia in inflammatory responses in the brain [11, 12]. We thus stained astrocytes in the hippocampus with anti-GFAP antibody, an astrocytic marker. In hippocampal regions (between CA1 region of Ammon's horn and dentate gyrus), the expression of GFAP was increased in the LPS-treated group (Figure 5(a)-IV) compared with that in the nontreated group (Figure 5(a)-I). The number of GFAP-positive cells in the 1.2 g/kg/day-treated group (Figure 5(a)-VII) and 2.4 g/kg/day-treated group (Figure 5(a)-X) was significantly less than that in the LPS-treated group, as shown in Figure 5(b).

We next examined the proinflammatory response in these regions of the 4 groups (nontreated group, LPS-treated group, 1.2 g/kg/day-treated group, and 2.4 g/kg/day-treated group) by using COX-2 immunohistochemistry, as COX-2 is well known to be an important enzyme that regulates LPS-induced inflammation [13]. In the nontreated group, only weak COX-2 immunoreactivity was detected (Figure 5(a)-II); but the immunoreaction was markedly stronger in the LPS-treated group (Figure 5(a)-V). In the 1.2 g/kg/day-treated group and 2.4 g/kg/day-treated group, the level of this immunoreactivity was reduced compared with that in the LPS-treated group (Figure 5(a)-VIII and XI, resp., and Figure 5(c)). In all groups, the GFAP-positive cells (astrocytes) were immunopositive for COX-2 (Figure 5(a)-III, VI, IX, and XII); but the microglia were not (data not shown). These results indicate that activated astrocytes contributed to COX-2 expression after* i.p.* administration of LPS, whose expression was attenuated by the dried peels of* C. kawachiensis*.

### 3.5. Effect of the Dried Flavedo and Juice on LPS-Induced Microglial Activation and COX-2 Expression

**Figure 6:**
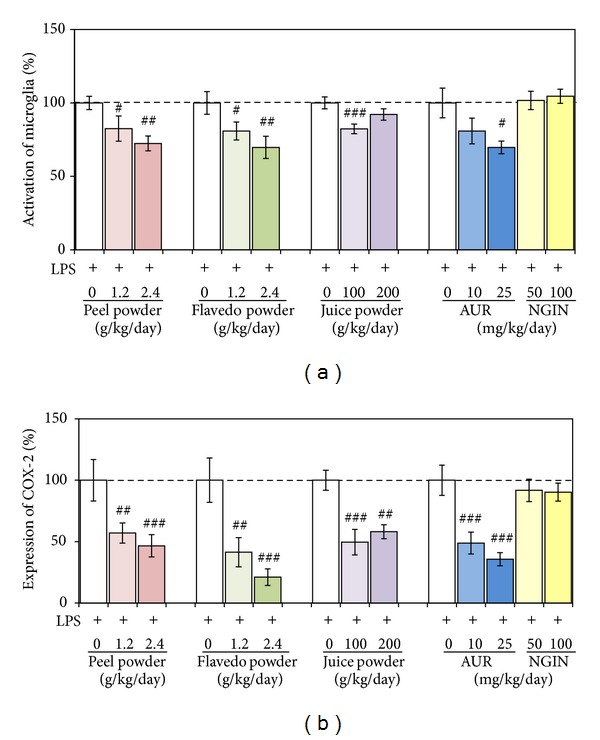
Effect of various samples derived from* C. kawachiensis* and of authentic NGIN/AUR on LPS-induced loss of LPS-induced microglial activation (a) and LPS-induced expression of COX-2 (b). Values are means ± SEM. Symbols indicate significant differences versus LPS-treated group (^#^ *P* < 0.05, ^##^ *P* < 0.01, and ^###^ *P* < 0.001).

We then administered the dried powder derived from flavedo or juice to LPS-injected mice. The amounts of each compound in these samples are indicated in Table 2. As was shown in Figure 4,* i.p.* administration of LPS induced microglial activation (***P < 0.001), and Figure 6(a) shows the effect of various citrus samples on the LPS-induced microglial activation taken as 100%: this activation was reduced significantly in the 1.2 g of peel/kg/day-treated group (^#^ *P* < 0.05), 2.4 g of peel/kg/day-treated group (^##^ *P* < 0.01), 1.2 g of flavedo/kg/day-treated group (^#^ *P* < 0.05), 2.4 g of flavedo/kg/day-treated group (^##^ *P* < 0.01), and 100 g of juice/kg/day-treated group (^###^ *P* < 0.001). Regarding their effect on COX-2 expression, Figure 6 shows the extent of COX-2 expression of sample groups versus COX-2 expression in the LPS-treated group taken as 100%: a significant reduction in expression was found in the 1.2 g of peel/kg/day-treated group (^##^ *P* < 0.01), 2.4 g of peel/kg/day-treated group (^###^ *P* < 0.001), 1.2 g of flavedo/kg/day-treated group (^##^ *P* < 0.01), 2.4 g of flavedo/kg/day-treated group (^###^ *P* < 0.001), 100 g of juice/kg/day-treated group (^###^ *P* < 0.001), and 200 g of juice/kg/day-treated group (^##^ *P* < 0.01). These results indicate that not only the peels of* C. kawachiensis* but also the flavedo tissue/juice had the ability to suppress LPS-induced inflammation in the brain.

### 3.6. Effect of NGIN or AUR on LPS-Induced Microglial Activation and COX-2 Expression

The primary compound in the dried peels of* C. kawachiensis* was NGIN (Figure 2 and Table 1), and the dose of NGIN for the 1.2 g of peel/kg/day-treated group and 2.4 g of peel/kg/day-treated group was equivalent to 52.8 mg/kg/day and 106 mg/kg/day, respectively (Table 2). These results suggested that the main candidate for the anti-inflammatory effect of the dried peels of* C. kawachiensis* was NGIN. We thus* s.c.* preadministered commercially obtained authentic NGIN to LPS-treated mice at the dose of 50 mg/kg/day and 100 mg/kg/day. As a result, NGIN could suppress neither the microglial activation (Figure 6(a)) nor COX-2 expression (Figure 6(b)), suggesting that the main candidate for the anti-inflammatory effect of the dried peels of* C. kawachiensis* was not NGIN.

We also examined the effect of* s.c.* administered AUR in the amount of 10 mg/kg/day or 25 mg/kg/day on LPS-induced inflammation in the brain. The dose of AUR in the 2.4 g of peel/kg/day-treated group was almost equivalent to that in the 10 mg of AUR/kg/day-treated group. As shown in Figure 6(a) and 6(b), the mice of the 25 mg of AUR/kg/day-treated group showed significantly suppressed inflammation (^#^ *P* < 0.05 for IBA-1-positive cells; ^###^ *P* < 0.001 for COX-2-positive cells). In the 10 mg of AUR/kg/day-treated group, IBA-1-positive cells tended to be reduced in number (*P* = 0.147); and COX-2 expression was still significantly (^###^ *P* < 0.001) suppressed even at this lower dose. Therefore, these findings suggest that the main candidate for the anti-inflammatory effect of the dried peels of* C. kawachiensis* was AUR.

## 4. Discussion

The present study showed that orally administered dried peels of* C. kawachiensis* could ameliorate LPS-induced inflammation in the mouse brain. This is the first report to demonstrate that citrus peel can suppress inflammatory responses in the brain.* C. reticulata *or* C. unshiu* peels are a stomachic and are employed as “chinpi” by practitioners of Chinese/Japanese traditional herbal medicine; and a recent paper showed that “chinpi” could inhibit the expression of inducible nitric oxide synthase (iNOS), COX-2, nitric oxide (NO), prostaglandin E_2_ (PGE_2_), tumor necrosis factor (TNF)-*α*, and interleukin (IL)-6 in LPS-stimulated RAW 264.7 cells, a macrophage cell line [14]. But the* in vivo* effect of “chinpi” on inflammation in the brain was not investigated.

The dried peel powder of* C. kawachiensis* contained a high amount of NGIN, middle amounts of NRTN and AUR, and a low amount of HMF (Figure 2 and Table 1). Although the major compound in the dried peels of* C. kawachiensis* was NGIN, the results obtained in this study together with our previous results suggest that the major functional compound within the dried peels of* C. kawachiensis* for causing the anti-inflammation effect in these model mice was AUR. The reasons for this conclusion are as follows: (1) an anti-inflammatory effect on the brain was also observed by the administration of authentic AUR given alone (Figure 6); (2) the administration of authentic NGIN alone had so much effect (Figure 6); (3) NRTN, being an isomer of NGIN, would also likely have no anti-inflammatory property. although we did not investigate the effect of authentic NRTN given alone in this present study; and (4) the amount of HMF in the dried peels of* C. kawachiensis* was too low to have exerted its effects.

With respect to NGIN, accumulating evidence indicates that it potently inhibits oxidative stress not only* in vitro* [15–17] but also* in vivo*. For example, in the latter case (1) the preadministration of NGIN (100 mg/kg/day for 7 days,* i.p.*) into ischemic model mice attenuates behavioral alterations and neuronal damage [18]; (2) the administration of NGIN (80 mg/kg/day for 7 days,* i.p.*) into model mice with kainic acid-induced epilepsy attenuates behavioral alterations and cognitive impairment [19]; (3) the administration of NGIN (80 mg/kg/day for 2 weeks,* p.o.*) into model rats with 3-nitropropionic acid-induced Huntington's disease suppresses neuronal apoptosis [19, 20]; (4) the administration of NGIN (40 or 80 mg/kg/day for 25 days,* p.o.*) to colchicine-injected rats attenuates the cognitive impairment in them [21]; (5) the administration of NGIN (40 or 80 mg/kg/day for 6 weeks,* p.o.*) into mice chronically treated with D-galactose lessens their cognitive impairment and mitochondrial dysfunction [22]; and (6) the administration of NGIN (100 mg/kg/day for 16 weeks) to Alzheimer's disease-model mice (APPswe/PS1dE9) improves long-term memory [23]. As NGIN and its metabolites have been shown to have difficulty in crossing the blood-brain barrier (BBB) [24], the BBB might have been destroyed in these cases, consequently permitting NGIN (and probably NRTN) to exert a potent antioxidative effect on the brain.

The most important question to be answered was whether (1) AUR passed through the BBB and acted directly in the brain as an anti-inflammatory agent or (2) AUR suppressed the peripheral inflammation, which was followed by the suppression of central inflammation. We suggested that a part of AUR, at least, could penetrate the BBB because (1) AUR is a hydrophobic small molecule; (2) our preliminary data showed that* i.p.* administration of AUR was useful to suppress inflammation induced by central administration of LPS (unpublished data). Inflammation in the brain is associated with various neurodegenerative diseases, such as Parkinson's disease and Alzheimer's disease [25, 26], as well as with ischemia and trauma [1]. Thus, the dried peels of* C. kawachiensis*, which contain high amounts of AUR, might be useful for the reduction of neuroinflammation-related brain diseases.

## 5. Conclusions

Upon pretreatment of LPS-injected mice with dried powder of* C. kawachiensis,* inflammatory responses in the brain were reduced. The dried powder derived from flavedo or juice of* C. kawachiensis* was also effective as an anti-inflammatory. The main component responsible for these effects was concluded to be AUR.

## Figures and Tables

**Table 1:** Amounts of AUR, HMF, NGIN, and NRTN in the dried peel, flavedo, albedo, and juice.

	AUR (mg/g)	HMF (mg/g)	NGIN (mg/g)	NRTN (mg/g)
Dried peel	4.07	0.27	44.02	4.46
Dried flavedo	9.62	1.37	13.91	3.97
Dried albedo	0.10	0.01	14.07	1.17
Dried juice	0.32	ND	1.58	0.68

**Table 2:** Doses of AUR, HMF, NGIN, and NRTN of each group.

	AUR (mg/kg/day)	HMF (mg/kg/day)	NGIN (mg/kg/day)	NRTN (mg/kg/day)
Peel				
1.2 g/kg/day-treated group	4.88	0.324	52.8	5.35
2.4 g/kg/day-treated group	9.77	0.648	106	10.7
Flavedo				
1.2 g/kg/day-treated group	11.5	1.64	16.8	4.76
2.4 g/kg/day-treated group	23.1	3.29	33.4	9.53
Juice				
100 g/kg/day-treated group	32.0	ND	158	68
200 g/kg/day-treated group	64.0	ND	316	136
